# Contribution of non-extensor muscles of the leg to maximal-effort countermovement jumping

**DOI:** 10.1186/1475-925X-4-52

**Published:** 2005-09-06

**Authors:** Akinori Nagano, Taku Komura, Shinsuke Yoshioka, Senshi Fukashiro

**Affiliations:** 1 Computational Biomechanics Unit, RIKEN; Hirosawa 2-1, Wako, Saitama, 351-0198, Japan; 2 Department of Computer Engineering and Information Technology, City University of Hong Kong; 83 Tat Chee Avenue, Kowloon, Hong Kong; 3 Dpartment of Life Sciences (Sports Sciences), the University of Tokyo; Komaba 3-8-1, Meguro, Tokyo, 153-8902, Japan

## Abstract

**Background:**

The purpose of this study was to determine the effects of non-extensor muscles of the leg (i.e., muscles whose *primary *function is not leg extension) on the kinematics and kinetics of human maximal-effort countermovement jumping. Although it is difficult to address this type of question through experimental procedures, the methodology of computer simulation can be a powerful tool.

**Methods:**

A skeletal model that has nine rigid body segments and twenty degrees of freedom was developed. Two sets of muscle models were attached to this skeletal model: all (most of) major muscles in the leg ("All Muscles" model) and major extensor muscles in the leg (i.e., muscles whose primary function is leg extension; "Extensors Only" model). Neural activation input signal was represented by a series of step functions with a step duration of 0.05 s. Simulations were started from an identical upright standing posture. The optimal pattern of the activation input signal was searched through extensive random-search numerical optimization with a goal of maximizing the height reached by the mass centre of the body after jumping up.

**Results:**

The simulated kinematics was almost two-dimensional, suggesting the validity of two-dimensional analyses when evaluating net mechanical outputs around the joints using inverse dynamics. A greater jumping height was obtained for the "All Muscles" model (0.386 m) than for the "Extensors Only" model (0.301 m). For the "All Muscles" model, flexor muscles developed force in the beginning of the countermovement. For the "All Muscles" model, the sum of the work outputs from non-extensor muscles was 47.0 J, which was 13% of the total amount (359.9 J). The quantitative distribution of the work outputs from individual muscles was markedly different between these two models.

**Conclusion:**

It was suggested that the contribution of non-extensor muscles in maximal-effort countermovement jumping is substantial. The use of a computer simulation model that includes non-extensor muscles seems to be more desirable for the assessment of muscular outputs during jumping.

## Background

Jumping motions have been investigated by many researchers in the field of biomechanics in an effort to understand the coordination of the human body during explosive activities. A maximal-effort jumping is a suitable subject for this purpose, as the objective of a maximal-effort jumping can be defined in a very straightforward manner: "jump up as high as possible". Therefore less inter-subjects and intra-subject variability of body coordination is expected. In addition, jumping motions play important roles in many athletic activities such as track and field, basketball and volleyball. Therefore it is practically valuable to understand the biomechanics of the human body during jumping.

Researchers have reported many valuable insights regarding maximal-effort jumping motions using two-dimensional computer modelling and simulation [1-5]. Typically in these studies, leg muscles that have a primary function of leg extension (e.g., the m. gluteus maximus, m. rectus femoris, hamstrings, mm. vasti, m. gastrocnemius, m. soleus) were included in the model. In other words, other leg muscles that have a different primary function (e.g., joint flexion, abduction/adduction, rotation etc.; "non-extensor muscles") often were not explicitly implemented. Although it is true that the motion of the leg is mostly extension during jumping, there is a possibility that these non-extensor muscles do contribute to a jumping performance because of their three-dimensional anatomical configuration. Especially, when looking at the location of the origin, insertion and via-points of most muscles, it is observed that three-dimensional vectors instead of two-dimensional vectors better represent their line of action [6,7]. Therefore it is valuable to investigate whether or not these non-extensor muscles of the leg make a substantial contribution to jumping performance. (Note that muscles whose primary function is not leg extension are called "non-extensor muscles" in this paper. This nomenclature does not imply that these muscles do not contribute to leg extension at all. In fact, as the human body is a linked segmental system, the activity of a muscle can affect the actions of multiple joints/degrees of freedom in the system [8]. This paper utilized this nomenclature for the sake of simplicity.)

For that purpose, it is likely that the use of a three-dimensional neuromusculoskeletal model instead of a two-dimensional model is more straightforward. Anderson and Pandy [9] expanded their research on jumping motions using a three-dimensional model. Nagano et al. [10] also constructed a three-dimensional musculoskeletal model of a human ancestor's body that can be scaled up to represent the musculoskeletal system of modern humans [11]. The purpose of this study was to evaluate the contribution of non-extensor muscles of the leg to maximal-effort countermovement jumping using a three-dimensional neuromusculoskeletal model.

## Methods

**Figure 1:**
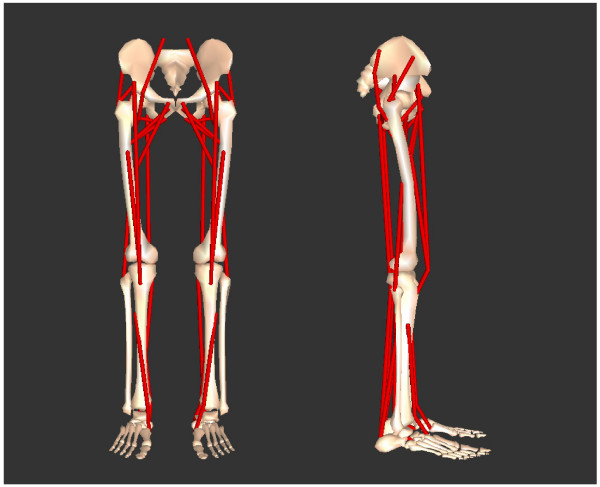
**The musculoskeletal model developed for this study. **The simulation model contained 9 rigid body segments, and the degrees of freedom of the model was 20.

A 3D neuromusculoskeletal model of the human body was constructed using DADS-3D (LMS CADSI, Coralville, Iowa, USA) with the FORTRAN-based USER.FORCE option. Detailed properties of this model have been reported in preceding studies [10,11]. The musculoskeletal model consisted of nine rigid body segments (the head-arms-trunk (HAT), right and left upper legs, right and left lower legs, right and left feet and right and left toes) connected with frictionless joints (Figure 1). Body segmental parameter values were derived from [12] (body mass = 73.1 kg). The hip joints were modelled as ball-and-socket joints that have three degrees of freedom. The knee joints were modelled as hinge joints. The ankle joints were modelled as universal joints [13]. The metatarsophalangeal joints were modelled as hinge joints with a tilted axis [7]. The total number of degrees of freedom of the model was 20.

The body was actuated by two different sets of muscles to construct the "All Muscles" model and the "Extensors Only" model. Thirty-two Hill-type lower limb muscles (16 muscles in each leg) were implemented in the "All Muscles" model (Table 1). These include all (more precisely, most of) major muscles found in a human leg. Fourteen muscles (7 muscles in each leg) were implemented in the "Extensors Only" model (Table 1). These include major leg extensor muscles only (i.e., muscles whose primary function is leg extension). Note that such biarticular muscles as the hamstrings, m. rectus femoris and m. gastrocnemius were regarded as extensor muscles. Under the joint configurations assumed during jumping, these muscles do develop more joint extension moments than joint flexion moments.

**Table 1:** The muscle parameter values used in this study. The values for each muscle are shown. F_max_: maximal isometric force of the contractile element. L_CEopt_: optimal length of the contractile element. α_pen_: pennation angle. L_slack_: slack (unloaded) length of the series elastic element. ILIOP: m. iliopsoas. GMAXI: m. gluteus maximus. GMEDI: m. gluteus medius. GMIN: m. gluteus minimus. ADDLO: m. adductor longus. ADDMA: m. adductor magnus. ADDBR: m. adductor brevis. HEXRO: merged hip external rotator muscles. RECTF: m. rectus femoris. HAMST: merged hamstrings. VASTI: mm. vasti. BFESH: m. biceps femoris short head. GASTR: m. gastrocnemius. TIBAN: m. tibialis anterior. SOLEU: m. soleus. OPFLE: merged monoarticular planter flexor muscles other than m. soleus. All of these muscles were implemented in the "All Muscles" model. Muscles whose primary function is leg extension are noted as "Extensor". Only these muscles were implemented in the "Extensors Only" model.

	F_max _(N)	L_CEopt _(m)	α_pen _(deg)	L_slack _(m)	
ILIOP	1544	0.104	8	0.130	
GMAXI	1883	0.142	5	0.125	Extensor
GMEDI	1966	0.054	8	0.078	
GMINI	849	0.038	1	0.051	
ADDLO	716	0.138	6	0.110	
ADDMA	1916	0.087	5	0.060	
ADDBR	531	0.133	0	0.020	
HEXRO	1512	0.054	0	0.024	
RECTF	1353	0.084	5	0.432	Extensor
HAMST	3054	0.080	15	0.359	Extensor
VASTI	6718	0.087	3	0.315	Extensor
BFESH	256	0.173	23	0.100	
GASTR	2044	0.045	17	0.408	Extensor
TIBAN	532	0.098	5	0.223	
SOLEU	5881	0.030	25	0.268	Extensor
OPFLE	3137	0.031	12	0.310	Extensor

Muscles investigated in [7] and [14] were considered for implementation. In order to perform computer simulation and numerical optimization within feasible computation time, it was necessary to limit the complexity of the model. Therefore muscles that have similar biomechanical function were merged to compose a single muscle group. For example, the m. vastus medialis, m. vastus intermedialis and m. vastus lateralis were merged as mm. vasti. Muscles or muscle groups whose maximal isometric force is greater than 500 N were selected. The m. biceps femoris short head, whose F_max _is smaller than 500 N, was also selected as the only mono-articular knee flexor muscle (Table 1). Coordinates of the origin, insertion and via-points of these muscles were derived from [7]. Muscle parameter values, i.e., the optimal contractile element length (L_CEopt_), maximal isometric force of the contractile element (F_max_), pennation angle (α_pen_) and unloaded length of the series elastic element (L_slack_), were derived from [7] and [14]. A specific tension value of 31.5 N/cm^2 ^[15] was utilized. A bilateral symmetry was assumed between the right side and the left side of the body.

**Figure 2:**
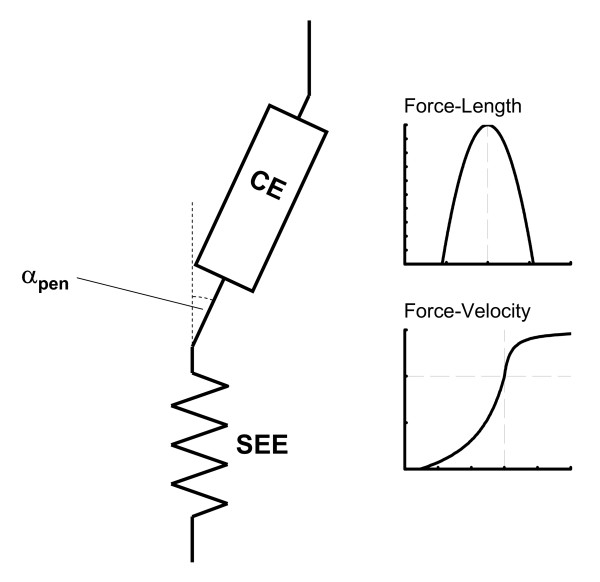
**The musculotendon model utilized in this study. **The musculotendon model was composed of a contractile element (CE) and a series elastic element (SEE). The effect of pennation angle (α_pen_) was also taken into consideration. The contractile element had the force-length-velocity relation, and the series elastic element had a non-linear force-length relation.

A muscle-tendon complex was composed of a contractile element (CE) and a series elastic element (SEE) serially connected with a pennation angle (α_pen_) (Figure 2). The mathematical model of the contractile element represented the force-length-velocity relations. Passive stress-strain property of the series elastic element was modelled with a quadratic function. A detailed mathematical representation of these components can be found in [16]. Neural activation input to individual muscles was represented by a series of step functions with duration of 0.050 s [17]. Excitation dynamics of the contractile element was modelled with a first-order ordinary differential equation as described in [18].

The interaction between the foot segments and the ground was modelled using the same form of equation as was reported in [9]. Passive joint properties that function to limit the joint range of motion were adopted from [9].

Maximal-effort countermovement jump motions were generated through computer simulation with the "All Muscles" model and the "Extensors Only" model. A simulation was initiated from an upright posture with the hip, knee and ankle joints slightly flexed (5 degrees: dorsiflexed for the ankle joint) to facilitate the generation of countermovement. Simulations were performed from exactly the same initial posture for these two models. Muscle activation input profiles were modified through Bremermann's numerical optimization [19] in which the jumping height was maximized. The optimal combination of the activation input profiles for the muscles was searched. The optimization process was terminated when the objective function value had not improved for 10,000 successive iterations, which corresponds to approximately 60,000 function evaluations without any improvement [11].

The instantaneous power output value of the contractile element (P_CE_) was calculated as the product of the force development (F_CE_) and the shortening speed (V_CE_; positive value for shortening and negative value for lengthening) of the contractile element:

P_CE _= F_CE_·V_CE _    (Eq. 1)

The work output of the contractile element (W_CE_) was calculated as the time integration of P_CE _from the start of simulation through the instant of take off:



## Results

The maximal height reached by the mass centre of the body measured from the floor was 1.317 m for the "All Muscles" model and 1.233 m for the "Extensors Only" model (Table 2). The jumping height measured from the starting posture was 0.386 m and 0.301 m, respectively. With the body mass of 73.1 kg and the gravitational acceleration of 9.81 m/s^2^, the energy gain of the mass centre of the body throughout the jumping motion was 277 J and 216 J, respectively.

**Table 2:** The results of the numerical optimization obtained in this study. H_init_: initial height of the mass centre of the body. H_max_: maximal height reached by the mass centre of the body. E_gain_: energy gain of the mass centre of the body through the jumping motion calculated from the jump height, body mass and acceleration due to gravity. Δ: difference between the values for the "All Muscles" model and for the "Extensors Only" model.

	H_init _(m)	H_max _(m)	Jump Height (m)	E_gain _(J)
All Muscles	0.931	1.317	0.386	277
Extensors Only	0.931	1.233	0.301	216
Δ	-	0.084	0.084	61

**Figure 3:**
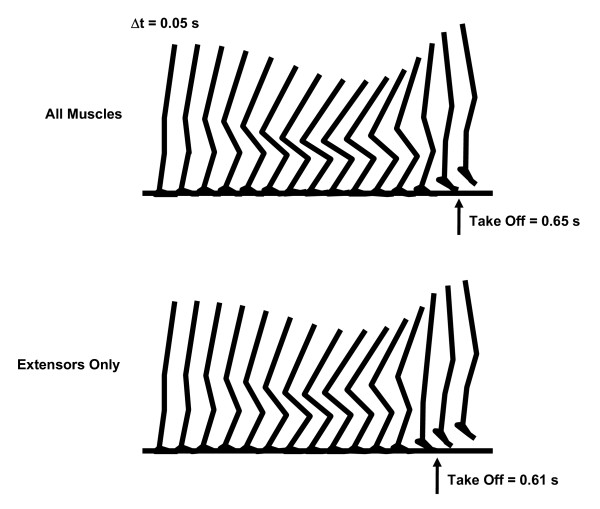
**The countermovement jumping kinematics generated in this study (sagittal view). **The take-off occurred 0.65 s and 0.61 s after the start of simulation for the "All Muscles" model and for the "Extensors Only" model, respectively.

**Figure 4:**
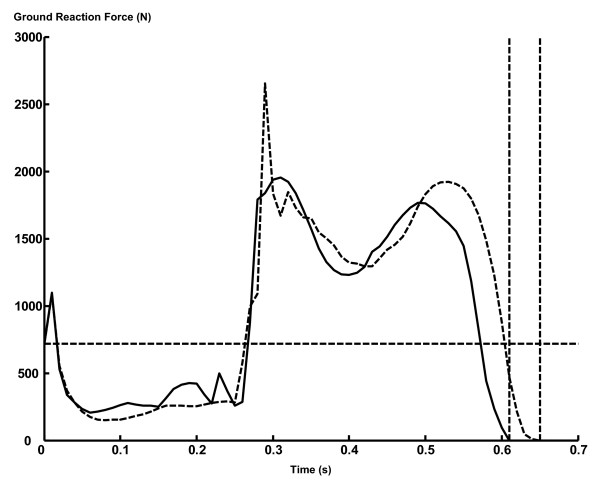
**The profile of ground reaction force. **The dashed curve represents the value for the "All Muscles" model, whereas the solid curve represents the value for the "Extensors Only" model. The dashed vertical lines represent the instant of take-off. The dashed horizontal line represents the body weight.

Realistic kinematics of jumping was generated both for the "All Muscles" model and for the "Extensors Only" model. Sagittal views of the kinematics are presented as Figure 3. The motions of the segments/joints outside of the sagittal plane were small (~10 deg; not shown), suggesting that the motion of the skeletal system was mostly two-dimensional. Take-off occurred at 0.65 s and 0.61 s after the start of simulation for the "All Muscles" model and for the "Extensors Only" model, respectively. Ground reaction force profiles are shown in Figure 4.

**Figure 5:**
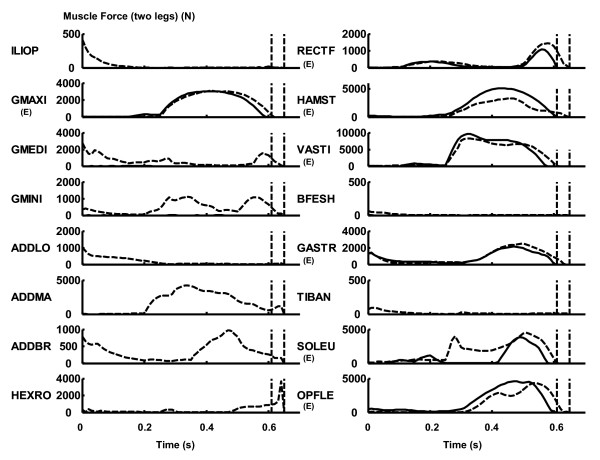
**The profile of muscle force output. **The dashed curve represents the value for the "All Muscles" model, whereas the solid curve represents the value for the "Extensors Only" model. The dashed vertical lines represent the instant of take-off. The added values for two contralateral muscles are shown. The muscles whose primary function is leg extension (Table 1) are noted by (E).

In the "All Muscles" model, joint flexor muscles such as the m. iliopsoas, m. biceps femoris short head and m. tibialis anterior were activated in the beginning of the countermovement phase (Figure 5). For the hamstrings, mm. vasti and other plantar flexor muscles, force output was greater for the "Extensors Only" model than for the "All Muscles" model. For the m. rectus femoris, m. gastrocnemius and m. soleus, force output was greater for the "All Muscles" model than for the "Extensors Only" model.

**Figure 6:**
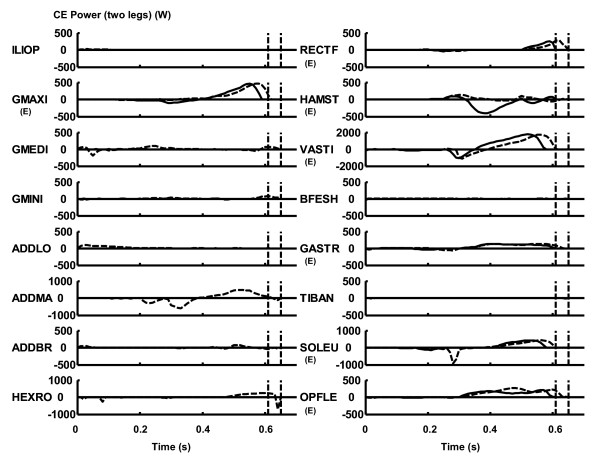
**The profile of the power output of the contractile element. **The dashed curve represents the value for the "All Muscles" model, whereas the solid curve represents the value for the "Extensors Only" model. Positive is concentric and negative is eccentric. The dashed vertical lines represent the instant of take-off. The added values for two contralateral muscles are shown. The muscles whose primary function is leg extension (Table 1) are noted by (E).

For the "All Muscles" model, non-extensor muscles such as hip adductors and external rotators performed relatively little work (Table 3, Figure 6), although the sum of the work outputs was substantial (47.0 J). The behaviour of the hamstrings was markedly different between the "All Muscles" model and the "Extensors Only" model. Specifically, for the "All Muscles" model, the hamstrings exerted relatively small magnitude of positive work suggesting that the action of this muscle was mostly isometric. On the other hand, for the "Extensors Only" model, this muscle had relatively large negative work suggesting that the action of this muscle was mostly eccentric (Table 3, Figure 6).

**Table 3:** The amount of the mechanical work performed by the contractile element. The integrated values from the start of simulation through the instant of take-off. The added values for two legs (two contralateral muscles) are shown. Δ: difference between the values for the "All Muscles" model and for the "Extensors Only" model.

	CE Work (two legs) (J)		
	All Muscles	Extensors Only	Δ
ILIOP	2.0	0.0	2.0
GMAXI	47.8	32.6	15.2
GMEDI	4.1	0.0	4.1
GMINI	4.5	0.0	4.5
ADDLO	10.3	0.0	10.3
ADDMA	9.1	0.0	9.1
ADDBR	2.4	0.0	2.4
HEXRO	14.8	0.0	14.8
RECTF	15.0	14.7	0.3
HAMST	10.1	-36.1	46.2
VASTI	133.1	172.2	-39.1
BFESH	0.4	0.0	0.4
GASTR	27.1	24.7	2.4
TIBAN	-0.6	0.0	-0.6
SOLEU	28.3	40.3	-12.0
OPFLE	51.5	39.4	12.1

SUM	359.9	287.8	72.0

## Discussion

The purpose of this study was to evaluate quantitatively the contribution of non-extensor muscles (muscles whose primary function is not leg extension) of the leg to maximal-effort countermovement jumping. Details of the simulation model utilized in this study have been described in [11]. In that study, a countermovement jumping motion simulated with the "All Muscles" mode have been analyzed and compared with the experimental data reported in preceding studies, and the validity of the modelling and simulation has been discussed. The optimized jumping height was smaller for the "Extensors Only" model than for the "All Muscles" model by 0.084 m (Table 2; 28.0%). In this study, the decrement in performance was caused by the absence of non-extensor muscles. This implies that non-extensor muscles do have substantial contributions to a maximal-effort countermovement jumping performance.

In both cases, the general characteristics of the jumping kinematics obtained through the numerical optimization process (Figure 3) were similar to the ones obtained through experimental data collection of human countermovement jumping [20], although the motion of the body and joint excursions were greater for the "All Muscles" model than for the "Extensors Only" model (Figure 3). Only limited motions of the skeletal system were observed outside of the sagittal plane in this study. It should be noted that the computer simulation model utilized in this study has a capability to perform fully three-dimensional motions (e.g., hip joint abduction/adduction etc.). Nonetheless, the simulation model chose to perform almost two-dimensional motions. This finding supports that the two-dimensional inverse dynamic analyses on jumping performed in numerous preceding studies are mostly valid. Especially when calculating such mechanical variables as net joint reaction forces, net joint moments and power outputs of joints, reliable calculations can be assumed.

When performing computer simulation of jumping, it is assumed to be acceptable to construct a two-dimensional skeletal model of the human body for the same reason. However, when attaching muscle models to the skeletal model, it will be more appropriate to explicitly consider the contribution of non-extensor muscles of the leg. Implementing three-dimensional configuration of these muscles will be the most straightforward solution. Calculating the projection of the line of action of these muscles to the sagittal plane will be another option to accomplish this.

Regarding the profiles of ground reaction force (Figure 4), two peaks were observed during the push-off phase. The first peak was mostly caused through the interaction between the heel and the floor in the beginning of the push-off phase, whereas the second peak was mostly caused through the interaction between the toe and the floor in the last part of the push-off phase. This profile of ground reaction force with two peaks is often observed in ground reaction force data collected from human subjects during a maximal-effort countermovement jumping [1,21]. The profile of ground reaction force was bumpy because each foot was modelled with only five contact points [11]. The profile will become smoother with more contact points in a foot, although this modification will greatly increase the computation time.

In the beginning of countermovement, joint flexor muscles, such as the m. iliopsoas, m. biceps femoris short head and m. tibialis anterior, developed force (Figure 5). This resulted in a greater countermovement for the "All Muscles" model than for the "Extensors Only" model. This seems to suggest that the contribution of these flexor muscles in the beginning of countermovement should be considered when investigating the mechanism of maximal-effort countermovement jumping motion. For the "All Muscles" model, non-extensor muscles such as the m. gluteus medius, m. gluteus minimus, adductors and hip external rotators had relatively minor individual contributions in terms of mechanical work and power outputs (Table 3). However, when the work outputs of these muscles were added together, the amount was substantial (47.0 J in 359.9 J; 13%), suggesting that the contribution of these muscles in jumping motion is not negligible.

For the m. gluteus maximus, m. rectus femoris, hamstrings, m. gastrocnemius and other monoarticular plantar flexor muscles (OPFLE), the work output was greater for the "All Muscles" model than for the "Extensors Only" model (Table 3). This result is very reasonable considering that the jumping height was greater for the "All Muscles" model than for the "Extensors Only" model (Table 2). Generally speaking, to achieve a higher jumping performance in an optimally-coordinated movement, muscles need to perform more work. As the jumping height was greater for the "All Muscles" model, greater mechanical outputs of muscles are reasonably expected for this model than for the "Extensors Only" model.

However, there were two exceptions; for the mm. vasti and m. soleus, the work output was greater for the "Extensors Only" model than for the "All Muscles" model. This result came from the fact that the "Extensors Only" model underwent a smaller countermovement compared to the "All Muscles" model (Figure 3, Figure 4). In this study, the amount of work output was calculated as a net (positive and negative) value from the start of a motion through the instant of take-off (Eq. 1 and 2). As the magnitude of countermovement (negative phase) was smaller for the "Extensors Only" model, the net amount of work output of the mm. vasti and m. soleus was calculated to be greater for this model. This phenomenon can be observed in Figure 6, where a smaller negative power output of these muscles during the countermovement is exhibited for the "Extensors Only" model. As the mm. vasti is a major knee extensor and the m. soleus is a major ankle plantarflexor, these muscles had to function to brake the downward momentum generated during the countermovement. This had the effect of reducing the net work output of these muscles.

For the hamstrings, a positive work output (10.1 J) was calculated for the "All Muscles" model, whereas a negative (-36.1 J) value was calculated for the "Extensors Only" model. It is observed that the contractile element of the hamstrings was mostly stretched in an eccentric manner in the "Extensors Only" model in the latter phase of the countermovement, resulting in a negative power (Figure 6) and work (Table 3) outputs. This is because only a few muscles that can act as extensors were available to brake the countermovement of the trunk segment in this model. Specifically, the m. adductor longus, m. adductor brevis and m. adductor magnus had been removed from the model. Therefore the inertial load of the trunk segment (moving downwards) imposed on the hamstrings in the latter phase of the countermovement was so great as to stretch this muscle in an eccentric manner, although this muscle was vigorously activated during this period (Figure 5). In other words, the hamstrings was not strong enough to brake the downward momentum of the trunk segment in a concentric manner. This discussion is consistent with the muscle force development profile shown in Figure 5. The force development of the hamstrings was greater for the "Extensors Only" model than for the "All Muscles" model (Figure 5), which is reasonable considering that the action of this muscle was mostly eccentric for the "Extensors Only" model (eccentric part of the force-velocity relation; Figure 2). On the other hand, as the "All Muscles" model had more muscles to function to brake the countermovement, the hamstrings could shorten itself and produce positive (concentric) work and power outputs. For example, the m. adductor magnus did function to brake the countermovement in this model (Figure 6). This result is consistent with the observation that there was a greater countermovement for the "All Muscles" model than for the "Extensors Only" model. As there were not enough muscles to brake the downward momentum generated during the countermovement, the optimal magnitude of countermovement for the "Extensors Only" model was smaller than that for the "All Muscles" model.

## Conclusion

As a result of this computer simulation study, it was found that the dynamics of the body motion is altered by the effects of non-extensor muscles. This finding is noteworthy considering that the overall kinematics of the body (Figure 3) and the ground reaction force profiles (Figure 4) were similar between the "All Muscles" model and the "Extensors Only" model. This result implies that it is desirable to consider explicitly the mechanical contribution of non-extensor muscles of the leg when investigating human jumping motions in terms of mechanical outputs of muscles. The use of a three-dimensional neuromusculoskeletal model seems to be more suitable for this purpose. However, results of this computer simulation study also supported that the nature of an optimally-coordinated countermovement jumping motion is mostly two-dimensional, which suggests the validity of the two-dimensional inverse dynamic analyses of net mechanical outputs around joints performed in many preceding studies.

## Authors' contributions

AN constructed the simulation model, performed computer simulation and other calculations, and drafted the manuscript. TK and SY participated in the process of model construction and numerical optimization. SF contributed valuable discussions and suggestions throughout this project, including the stage of manuscript writing. All authors read and approved the final manuscript.
